# The Need for Specific Penalties for Hacking in Criminal Law

**DOI:** 10.1155/2014/736738

**Published:** 2014-06-16

**Authors:** Sangkyo Oh, Kyungho Lee

**Affiliations:** Center for Information Security Technologies (CIST), Korea University, Seoul 136-701, Republic of Korea

## Abstract

In spite of the fact that hacking is a widely used term, it is still not legally established. Moreover, the definition of the concept of hacking has been deployed in a wide variety of ways in national literature. This ambiguity has led to various side effects. Recently in the United States, reforms collectively known as Aaron's Law were proposed as intended amendments to the Computer Fraud and Abuse Act (CFAA). Most experts expect that this change will put the brakes on the CFAA as a severe punishment policy, and result in a drop in controversial court decisions. In this study, we analyze the definitions and the penalties for hacking for each country and compare them with the national law and then make suggestions through more specific legislation. We expect it will reduce legal controversy and prevent excessive punishment.

## 1. Introduction

Hacking [1, 2] began as a way to find computer network security vulnerabilities in order to solve these problems and prevent malicious actions. The term “hacking” was used for the first time in the late 1950s in the minutes of a meeting of the Tech Model Railroad Club at the Massachusetts Institute of Technology (MIT). The original meaning of “hack” is just to feel pleasure in the work process itself. However, this meaning was gradually turned into a bad one through its constant association with computer criminals [3–5]. In other words, some hackers began to profit from the information that was pulled out of someone else's computer by breaking into it. Hackers also spread malicious programs through a computer network in order to destroy data. Some prefer to differentiate hackers—people who do not use a system illegally but expose holes within systems—from crackers—people who destruct systems. In general, however, distinguishing between hackers and crackers is meaningless to criminals.

Recently, Aaron Swartz who was the founder of Reddit and Demand Progress committed suicide. In early 2011, he hacked JSTOR, the paid journal database, using MIT's network. Federal prosecutors charged him with the maximum penalty of $1 million in fines, 35 years in prison, and asset forfeiture.

**Figure 1:**
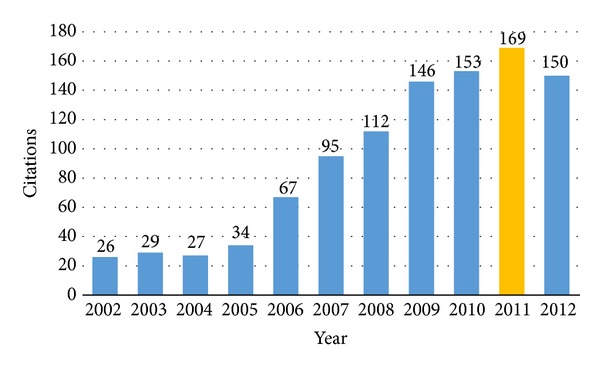
Computer Fraud and Abuse Act (CFAA) in the courts [9].

The Computer Fraud and Abuse Act (CFAA) [6–8] has been widely abused by prosecutors to hamper security research, to stifle innovation, and to lock people who have caused little or no economic harm away for years (Figure 1). The CFAA was originally intended to cover the offence of hacking in relation to defense and bank computers, but it has been expanded in order to cover every virtual computer on the Internet to mete out disproportionate penalties for virtual crimes [9].

In USA, reforms collectively known as Aaron's Law [10] intended as amendments to the CFAA have been proposed. The major proposed revisions to the CFAA are related to the use of the provisions “exceeds authorized access” and “access without authorization.” Punishment will be administered only if one or more technical or physical measures are intentionally bypassed. Furthermore, in terms of the penalty, the person will be punished only if the information obtained by hacking into a computer is valued over $5000. This change will put the brakes on the CFAA as a severe punishment policy, bring clarity, and reduce legal controversy in court decisions.

According to the legal provisions of South Korea, hacking means an act that unauthorized or authorized people use to abuse their authority to break into an information network by using an information processing device such as a computer. In other words, the current “Promotion of Information and Communications Network Utilization and Information Protection Act” is the same as the CFAA in USA just before its revision. Any person that violates this could be sentenced to less than three years' imprisonment or a fine of 30 million won or less. However, as in the case of Aaron Swartz, it has the potential to lead to an excessive application of legal principles.

As a result, this study analyzes the international justice and punishment for hackers, then compares them with the “Promotion of Information and Communications Network Utilization and Information Protection Act” through specific legislation related to judicial interpretation, and attempts to reduce legal controversy. Subsequently, we propose measures to prevent excessive punishment.

## 2. Penalties of Countries

In this study, we deal with the meaning of cybercrime [11] related to provisions of the law and analyze the principles common to the laws and penalties. We will use comparative law methods in a narrow sense. Two or more social systems and legal systems of the country will be compared. In addition, we will perform a comparative analysis of the contents of several laws for legislation or amendments.

Countries are selected by a specific rule based on the data collection possibility unity for analysis and effectiveness. Target countries are the USA, Germany, and China. USA recognizes cybersecurity as the national security dimensions of cybersecurity awareness. In the German legal system cybersecurity legislation has traditionally had the most profound influence on us [12]. Recently, there was a discussion about cybersecurity in China.

We will find the better way forward to amend the Promotion of Information and Communications Network Utilization and Information Protection Act by comparing it to the laws of each country.

### 2.1. Penalties of Germany

Information network for “electronic residential intrusion” penalties in Article 202(a) of the German Penal Code is provided. Information network intrusions method means to access protected information without the permission of the constituent elements or to allow a third party to access to information. The privacy protection provisions have penalty functions, so that no matter what is the information content of the object that has been breached, the act itself will be regarded as a crime. The penalty would not need to be a result of the breaches.

There is no restriction on how the system is used to bypass the security holes, even if access to the information you enter in a position to recognize the crime is established. If a person finds out a system password using trojans and phishing techniques, even if he is the owner of the corporation, it is considered a crime because it was done without the approval of the owner.

Constituent elements of the “access” mean the content of information that can be recognized. Thus, using the login information to access the network does not constitute a crime, so you are not subject to criminal penalties. However, direct access means that the information is recognizable. So, another way to find out the information is not appropriate.

### 2.2. Penalties of the USA

Computer hackers in the USA go to jail for 10 years for a first offence, and a recidivist gets up to 20 years in prison. In addition, any attempt to cause damage to computers will result in serious problems. Even if there is no explicit damage, the attempt to cause damage to computer would be punished by the legislative provisions. The scale of damage is estimated by the sum of the overall damage in one year. In particular, defense or national security cases can be punished without proof.

Causing damage through the use of computer malware, programs, information dissemination, and unauthorized computer intrusion have resulted in legislative provisions being introduced in the federal Criminal Code criminalizing. Distributed denial of service (DDoS) attacks also punished by federal Criminal Code and imposed penalties for cybercrime, such as hacking and viruses. The cybercrime sentencing standard has been tightened. If the cybercrime committed was intentional, it could result in up to 20 years in prison. Moreover, if damage to human life was caused, it could lead to life imprisonment.

The Cyber Security Enhancement legislation in 2002 (Cyber Security Enhancement Act of 2002) has introduced privacy protection, computer crime sentencing detail, and guide for enhanced penalties. Specifically, the guidelines were modified to consider the seriousness of the sentencing under Article 225 of the Computer Fraud and Abuse Act (Computer Fraud and Abuse Act, CFAA). Besides, a cyberattacker who intentionally or inadvertently violates the law and causes serious injury may go to jail for up to 20 years. In addition, intentionally or negligently causing death may be punished by life imprisonment.

### 2.3. Penalties of China

Cybercrimes are not regulated by one single special law in China. Rather, they are covered by a scope of laws and regulations with a comprehensive nature, such as Ordinance for Security Protection of Computer Information System, Criminal Law Articles 285–287, Decision Regarding the Maintenance of Internet Security, and Provisions on Administrative Punishment concerning the Management of Public Security [13].

In 2009, the Amendment to the Criminal Law of China (VII), which was deliberated at the 7th meeting of the Standing Committee of the Eleventh National People's Congress, was passed. Subsequently, China added “hacker” to the Criminal Code in order to be able to legally punish hacking. According to Article 285 of the existing criminal law in China, on the violation of state regulation and intrusion of national affairs, defense, construction, science, and technology, the area of unlawful breaching of computer information systems was punishable with less than three years of imprisonment.

However, under the existing laws, law enforcement agencies faced many challenges to be able to arrest “hackers.” They illegally intrude into someone else's account, computer system, and steal information such as passwords. There is also large-scale illegal control of another person's computer. Thus, they make a critical impact on network security. To ensure the correction of the insufficient legal grounds, the Criminal Code Amendment (7) Law was passed, while a second clause and third clause were added to Article 285 of the Criminal Code.

In addition, in 2011 the Supreme People's Court prepared for a trial to punish the people who unlawfully breached the network to obtain information or plant malware by “interpretation of the law for computer information criminal case.” The Supreme People's Procuratorate pointed out that the illegal market for the buying and selling of materials and tools for hacking was growing and it was regarded as a criminal offence. Previously, this was punishable with only three years in prison. A relatively light punishment was imposed. However, after this trial, it was held that acts such as providing software to hackers are grave criminal offences. Perhaps this “indirect” law is subject to the Criminal Code and it will allow those who commit this offence to be jailed for up to ten years.

## 3. Comparison of Criminal Laws

In this case, Republic of Korea court will be able to make a decision according to the “Promotion of Information and Communications Network Utilization and Protection Act.” Thus, violators would be punished by either a fine of up to 30 million won or a maximum prison sentence of three years [14, 15].

In addition, the United States court will be able to make a decision according to the copyright laws. At this time, a sentence of less than three years' imprisonment or a fine of 30 million won or less is applied.

Under German law, criminals are punished under Article 202a of the Criminal Code. They are sent to jail for less than three years. Also, pursuant to Article 109 of the Copyright Act, the punishment becomes less than three years in prison. On the other hand, China court can make an order to stop using it and may request damage compensation.

Punishment after the structuring of German cybersecurity legislation enables the integration of management. In the case of Korea, cybersecurity criminal penalties for infringement of the Information and Communication Network Utilization and Information Protection Act, the Criminal Code, E-Trade Promotion, Information Infrastructure Protection Act, Communications Privacy Act, and other laws and regulations are decentralized. It leads to problems with understanding penalties, and it makes it difficult to evaluate the laws.

In addition, the German cybersecurity legislation on cybersecurity violations and possible penalties are lumped together. Therefore, it is easy to understand the information. This leads to an effective general prevention of cybercrime. Furthermore, in terms of equality of penalties for legal regulations, it seems to be more preferable than distributed case. Laws and regulations are varied; the purpose of each and the operating policies are different. As a result, it is difficult to secure equity through consistency as a legal basis. It will be useful that law enforcement agencies who enforce the law (prosecutors and courts) interpret and apply the law with regard to the penalties in the Criminal Code.

In the case of Germany, the regulations do not disperse. Therefore, it is possible to punish without exception. But, in the case of Korea, there is no provision for punishment. We shall refer to the German legislative system. Penalties for and violations of cybersecurity provisions should be both included in the Criminal Code. This gap should be complementary. In this respect, the German postpunishment cybersecurity legislation is very useful to us. The main direction of the maintenance of the laws and regulations should be on the basic law. Furthermore, the provisions of other laws that are passed are too specialized or simplified, and common details should be defined in the basic laws.

Cybercrime in the USA began in the mid-1980s. The laws on cybercrime were made and developed through the interactions. However, the extent of the actual low level of criminal penalties was, in the 2000s, caused by the awareness of the seriousness of the damage that can arise and the strict punishment that can be imposed for such an offence. USA is constantly expanding the range of penalties depending on the gravity of the crime; however, its stringent sentencing of Aaron Swartz led to unfortunate side effects such as his suicide. This result is a good lesson for us on criminal law (Table 1).

## 4. Case Studies

The Ministry of Information and Communication announced amendments to the “Promotion of Information and Communication Network Utilization and Information Protection Act” as part of its follow-up measures to the “1.25 Internet Security Incident,” and to expand the scope of the penalty for cybercrime. Just an attempt at hacking or introduction of a virus can result in a criminal penalty with a maximum sentence of five years in prison or a fine of 50 million won.

### 4.1. Port Scan

**Figure 2:**
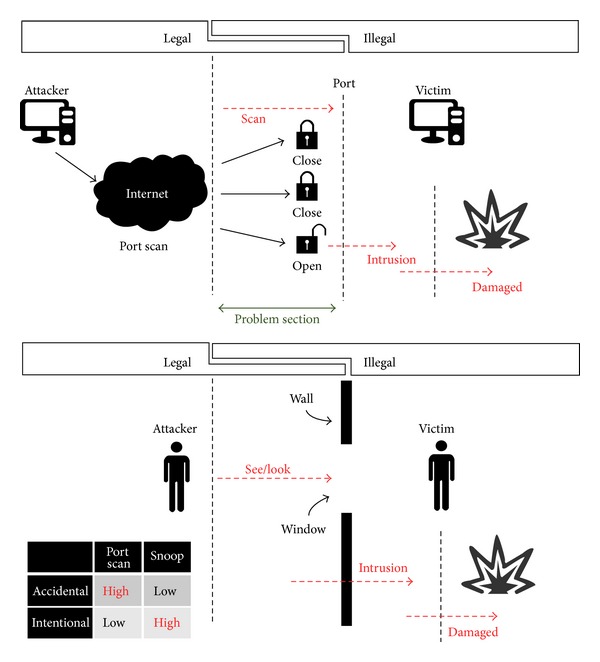
Legal comparison between port scan and snoop.

A port scan [16] is a subject of punishment in Korea because it is regarded as an attempt to attack. Strictly speaking, a port scan is a vulnerability inspection skill rather than a hacking attack. But sometimes hackers misuse such a skill to find out the host's weak point, and hackers try attacks based on this information (Figure 2).

This kind of hacking is considered a “trial of intrusion” rather than “intrusion.” However, it is an action “beyond the limits of authority” allowed and can be admitted as starting to execute an attack. Therefore, it can be punishable under Article 48 of the “Promotion of Information and Communication Network Utilization and Information Protection Act.”

But as mentioned earlier, focusing on “intrusion,” we can have other constructions of law. “Intrusion” means that the agent does not follow the normal certification procedure for utilizing the resource of information network system or uses an abnormal method to get authorization for entering information network system. When the resources of the information network system can be used arbitrarily, the resulting state is defined as completion of intrusion. Therefore, port scanning by hackers is defined not as the action of intrusion into an information network system but as the action of preparation for attempting to break into a targeted web server. We should regard the installing of a program for intrusion, when security vulnerability is discovered after port scanning, as the onset point of the execution of a hacking.

In addition, just executing a port scan does not damage the system. Actually, one can tell when a port scan is done on purpose by the periodicity or the specific port range of the object of port scanning. The malicious packets are usually filtered through a FW (firewall) or IDS (intrusion detection system).

### 4.2. Collecting Email Addresses

Similarly, there is an act concerning the collection of e-mail addresses. To punish this kind of preliminary act for spam mail sending is unreasonable and prior criminalization because it is unclear whether spam mail sending is a crime that warrants sentencing. We do not criminalize unwanted postal mail or leaflets that are delivered to a receiver in the real world. In this situation, the criminalization of spam mail is an unreasonable action. Moreover, there is no legal provisions to punish the collection of e-mail addresses what is not using some program or technical device [17].

### 4.3. iPhone Jail Breaking

It belongs to a hack that manipulates the kernel of operating system in hardware such as iPhone for using more than the originally programmed functions. It is a real interesting mix of professionals looking at this. But it is not violating copyright laws. Moreover, it is considered as having no intention to cybercrime. This pseudohacking is ruled out of subject to criminal prosecution [18].

### 4.4. Attempted DDoS Attack

DDoS attacker is punished by the law regarding the promotion of information and communication network use and protection of information Articles 48 and 71. But this law cannot punish an attempted crime. So DDoS Attacker will not be punished if there are no breakdowns in network. Moreover, the scope of attempted DDoS attack will be expanded by technological development [19].

## 5. Punishment Criteria

Determination of punishment means that decision punitive measures about convicted person [20]. At this time, determination of punishment should ensure predictability and controllability by the provisions.

Therefore, we need to adjust the scope of punishment for hacking as shown in Tables 2 and 3. As stated in Table 3, we should be able to punish legally with more detailed criteria.

At first, we divide the subject of punishment into two groups, with intention or without intention (inspection, just an attempt, etc.). We do not suggest concrete sentences but we need to apply different standards according to the scale of damage arising from the use of a malicious program.

## 6. Conclusions

Most cyberhackings are perpetrated by hackers to show off or satisfy themselves. Therefore, enhancing punishment is not the best way to prevent hacking. It is more important that they are educated about the damage caused by cyberhacking rather than punishing them. This will fundamentally solve the legal problem of preventing hacking attempts.

As we mentioned above, in USA, the penalties for cybercrime can be more than 20 years' imprisonment, but the definition of hacking is still not clear. Of course, penalties for hacking are also unclear due to the rapid development of technology. On the other hand, the domestic law for hackers provides a variety of penalties, and it is also not clear. This difference comes from the interpretation. Even if the user simply breaks a contract, unreasonable punishment is likely to be administered. Thus, it is necessary to define rules more clearly and specifically. Taking into consideration the relative uniformity, and the specific provisions of the criminal law in Germany, we must modify the “Promotion of Information and Communications Network Utilization and Information Protection Act” and other regulations. By doing this, legal controversy and excessive punishment will be reduced.

Additionally, reasonable adjustment of statutory punishment is needed in the future. At present, statutory punishment is distinguished from sentencing in current Korean court [21]. Sentencing level converged on the lowest limit of statutory punishment.

## Figures and Tables

**Table 1:** Comparing each country's criminal law.

	Korea	Germany	USA	China
Legislation	Network Act, 48	Criminal Law, 202(a)	CFAA (18 U.S.C 1030)	Criminal Law, 285
Criterion for punishment	Access abusing their authority	Access without authorization	Exceeds authorized access	Intrusion actions
Penalty (imprisonment)	3 years	3 years	Over 10 years	3–7 years
Feature	In distributed laws	In Criminal Law	Severe punishment	Punish acts of indirect

**Table 2:** The punishment criteria of hacking in Korea.

Action	Legal	Penalty	Intentional
Intrusion	No	Imprison/monetary	Yes/no

**Table 3:** The improved punishment criteria for hacking.

Action	Legal	Penalty	Intentional
Find out vulnerabilities	Yes	Fine	No
Attempted access	No	Monetary	Yes
Install malicious program	No	Imprisonment/monetary	Yes
Damage less than 50 million won by installing a malicious program	No	Imprisonment/monetary	Yes
Damage more than 50 million won by installing a malicious program	No	Imprisonment and monetary	Yes
